# Expression Profiling of Cellular MicroRNA in Asymptomatic HBsAg Carriers and Chronic Hepatitis B Patients

**DOI:** 10.1155/2017/6484835

**Published:** 2017-08-23

**Authors:** Xianliang Hou, Yan Liang, Jianing Chen, Yingfeng Wei, Ping Zeng, Lin Wang, Chong Lu, Hongyan Diao

**Affiliations:** State Key Laboratory for Diagnosis and Treatment of Infectious Diseases, Collaborative Innovation Center for Diagnosis and Treatment of Infectious Diseases, The First Affiliated Hospital, College of Medicine, Zhejiang University, Hangzhou 310003, China

## Abstract

**Background:**

MicroRNAs (miRNAs) may serve as potential molecular markers to predict liver injury resulting from chronic hepatitis B (CHB). In the present study, we want to study the expression profile and clinical significance of miRNAs at different stages of CHB virus infection.

**Methods:**

Using miRNA microarray, we investigated the global expression profiles of cellular miRNA in asymptomatic hepatitis B antigen carriers (ASCs) and CHB patients, compared with healthy controls (HCs).

**Results:**

We identified 79 and 203 differentially expressed miRNAs in the peripheral blood mononuclear cells of ASCs and CHB patients compared to HCs, respectively. Some of these miRNAs were common to ASCs and CHB patients, but another set of miRNAs that showed differential expression between ASCs and CHB patients was also identified. Gene ontology and pathway enrichment analysis showed that the target genes of the identified miRNAs played a role in important biological functions, such as learning or memory, cell-cell adherens junction, ion channel inhibitor activity, TGF-beta signaling pathway, and p53 signaling pathway.

**Conclusion:**

We identified some significant differentially expressed miRNA in different phases of HBV infection, which might serve as biomarkers or therapeutic targets in the future.

## 1. Introduction

Hepatitis B virus (HBV) is a hepatotropic noncytopathic DNA virus that is a major cause of liver diseases [1]. Eradication of HBV infection remains a global health challenge. More than 350 million people worldwide are persistent carriers of HBV, and many may progress to chronic liver disease. One to two million people die annually worldwide from HBV-related disease [2], which results in an increase in healthcare cost and other socioeconomic burdens. In most adults, HBV infection is self-limiting and characterized by quick viral clearance; however, in some cases, the patients become carriers or develop chronic persistent infection. According to their serological profile [3], patients can be divided into two well-distinguished subsets of subjects: (1) asymptomatic HBV carriers (ASCs) and (2) chronic hepatitis B (CHB) patients. ASCs show long-lasting inhibition of viral replication with viral load levels that are usually below 2,000 IU/mL and no biochemical, ultrasonographic, or histological evidence of liver injury. On the contrary, anti-HBe-positive CHB patients have active liver disease with a high risk of progression toward cirrhosis [4]. The difference in the responses to HBV infection is probably related to the exclusive dependence of HBV on host cellular machinery for its propagation and survival. Therefore, investigation of the interactions between HBV and host cells is crucial for understanding viral pathogenesis and the development of new antiviral therapies.

MicroRNAs (miRNAs) are small noncoding RNA molecules that are about 22 nucleotides long and regulate gene expression by base pairing with the 3′-untranslated region of target mRNAs, which usually leads to mRNA degradation or translational silencing. miRNAs have been identified in most types of cells and tissues and are involved in a variety of biological processes, such as inflammation, cell proliferation, development, differentiation, apoptosis, and tumorigenesis. Further, miRNAs play vital roles in the pathogenesis of various diseases, such as cancers and viral infections, through posttranscriptional regulation of more than 30% of human genes [5]. Cellular miRNAs also affect virus replication and pathogenesis, as demonstrated in the case of the liver-specific miRNA miR-122, which is essential for the replication of hepatitis C virus [6]. In addition, Zhang et al. found that the plasma miRNA profiles can indeed be used as a predictor of early virological response to interferon treatment in CHB patients [7]. In line with these findings, some reports suggest that circulating miRNAs may serve as potential molecular markers of liver injury resulting from CHB [8–10]. As the viral titer in the body, the degree of liver damage, and the immune characteristics vary between ASCs and CHBs, the expression profiles of miRNAs may also differ between these two groups of patients. However, there is not much information available about the relationships between cellular miRNAs and the different phases of chronic HBV infection. Therefore, the present study was conducted with the aim of filling in this gap in information.

Using miRNA microarray and PCR analysis, we investigated the global expression profiles of cellular miRNAs in peripheral blood mononuclear cells (PBMCs) from ASCs and CHB patients and identified a few novel miRNAs that were closely involved with the pathogenesis of HBV infection. Further, network analyses were used to determine the biological roles played by the target genes of these miRNAs in both ASCs and CHB patients.

## 2. Material and Methods

### 2.1. Clinical Samples

Human blood samples were obtained from healthy donors and patients with their informed consent. The study group included sixteen ASC patients, sixteen CHB patients, and sixteen healthy controls (HCs), who were recruited from the First Affiliated Hospital, College of Medicine, Zhejiang University, between 2014 and 2015. The study protocol conforms to the ethical guidelines of the 1975 Declaration of Helsinki, and all the patients provided their written informed consent for participation. The symptoms of all the patients were diagnosed according to the previously described criteria [11]. The level of HBs antigen was measured quantitatively using the Abbott chemiluminescence immunoassay kit (Abbott Japan, Tokyo, Japan). Quantitative detection of HBV DNA was performed using an ABI7300-type quantitative PCR instrument (Applied Biosystems, USA). The results are expressed as the number of log10 international units per milliliter.

### 2.2. Separation of PBMCs, RNA Extraction, and miRNA Microarray

Samples containing 5 ml of blood were collected. PBMCs were isolated using standard density-gradient centrifugation on Ficoll-Paque (Amersham Biosciences, Freiburg, Germany). Total cellular RNA from the PBMCs was extracted using the TRIzol RNA reagent (Invitrogen, Carlsbad, CA, USA) according to the manufacturer's instructions. Low-molecular-weight RNA was isolated using the mirVana miRNA isolation kit (Ambion, Austin, TX, USA) [12]. We performed miRNA microarray analysis to identify HBV-associated differences in cellular miRNA profiles between three ASC patients and three CHB patients and three healthy controls. miRNA microarray analysis was performed using the miRMAX microarray at KangChen Bio-Tech Corporation (Shanghai, China) [13]. The arrays were scanned using an Axon GenePix 4000B microarray scanner, and GenePix pro V6.0 was used to read the raw intensity of the image. We used the median normalization method to obtain normalized data (normalized data = [foreground value – background value]/median, where the median is the 50 percent quantile of microRNA intensity, which was greater than 30 in all the samples after background correction). An miRNA was considered to be differentially expressed if the difference in its expression was more than 2.0-fold.

### 2.3. Quantitative Real-Time PCR for miRNA Verification

The quantitative PCR validation group consisted of 16 CHB patients, 16 ASC patients, and 16 healthy controls. Four differentially expressed miRNA were randomly selected for verification. Expression of these mature miRNAs was assayed using the Mir-X miRNA First-Strand Synthesis Kit (number 638313, Clontech Laboratories, Inc.) and SYBR Premix Ex Taq II (Tli RNase H Plus) kit (number RR820A, Takara Bio, Inc.). Briefly, in the Mir-X cDNA synthesis reaction, RNAs are poly(A)-tailed using poly(A) polymerase and then copied using a modified oligo(dT) primer and SMART MMLV Reverse Transcriptase. The reaction mixture contained 5 *μ*l of 2× mRQ Buffer, 3.75 ul RNA sample (0.25–8 ug), and 1.25 ul mRQ Enzyme. The reaction was performed as follows: 1 hour at 37°C and then terminate at 85°C for 5 min to inactivate the enzymes. These synthetic cDNA were then specifically and quantitatively amplified using a miRNA-specific primer and SYBR advantage qPCR chemistry. All the primers for quantitative PCR (shown in Table 1) were synthesized by Sangon Biotech (Shanghai) Co., Ltd. The reaction mixture contained 5 *μ*l of 2× SYBR Premix Ex Taq II (Tli RNase H Plus), 0.2 ul miRNA-specific primer (20 uM), 0.2 ul mRQ 3′ primer, 0.2 ul ROX Reference Dye (50X), and nuclease-free water, which made a total volume of 8 *μ*l. Each 8 *μ*l mixture was added to a well in a 384-well PCR plate, and this was followed by addition of 2 *μ*l cDNA in each hole. The reaction conditions were as follows: 95°C for 2 min followed by 40 cycles of 95°C for 15 s and 60°C for 45 s [14]. The relative amount of miRNA was normalized against an internal control, U6 snRNA, and the fold change in the amount of each miRNA was calculated using the 2^−ΔΔCT^ method.

### 2.4. Gene Ontology Analysis and Signaling Pathway Analysis

Target genes of the differentially expressed miRNAs were predicted based on agreement between the two databases to predict miRNAs' target genes: targetscan7.1 and mirdbV5. Signaling pathway analysis and gene ontology (GO) analysis were performed to determine the biological functions of the target genes of differentially expressed miRNAs. The genes were mapped to each node in the GO database, and the number of genes in each node was calculated. Signaling pathway analysis is a functional analysis for mapping genes to KEGG pathways. GenMAPP v2.1 was used to map the genes to the KEGG database through the signaling pathway, and then statistical analysis was performed to determine the degree of enrichment of the genes in each pathway [15].

### 2.5. Statistical Analysis

Data analysis was performed using SPSS 19. Data were expressed as the mean ± standard deviation values. The statistical significance of differences between two groups was analyzed by the *t*-test.

## 3. Results

### 3.1. Patient Characteristics

The clinical characteristics of the patients according to group are shown in Table 2. The mean age and male : female ratio were similar between the three groups. The distribution of ALT and AST was not found to be different between ASC group and HC group, but it is significantly increased in CHB group compared with ASC group and HC group. The average amounts of HBV DNA were higher in CHB than in ASC, but there was no significant difference. The prevalence of hepatitis B surface antigen (HBsAg) was 62.5% in CHB and 25% in ASC. There was significant difference between them (*P* = 0.033).

### 3.2. miRNA Expression by Microarray Analysis

Among the 2077 human mature miRNAs investigated by the arrays, 79 miRNAs were found to be differentially expressed (>2-fold) between the ASC group and HC group, which accounted for 3.80% of all the miRNAs investigated. Of these, 11 were upregulated and 68 were downregulated in the ASC group. In addition, 203 miRNAs (9.77%) were differentially expressed (>2-fold) between the CHB group and HC group, including 118 upregulated miRNAs and 85 downregulated miRNAs in the CHB group. Further, 144 (6.93%) miRNAs exhibited more than a 2-fold difference in expression between the CHB group and the ASC group, of which 115 miRNAs were upregulated and 29 were downregulated. Among the differentially expressed miRNAs, 38 miRNAs were common to both the ASC group and CHB group, 5 of which were upregulated and 33 of which were downregulated in both groups (Table 3).

### 3.3. Validation of the Microarray Results by Quantitative Real-Time PCR

**Figure 1:**
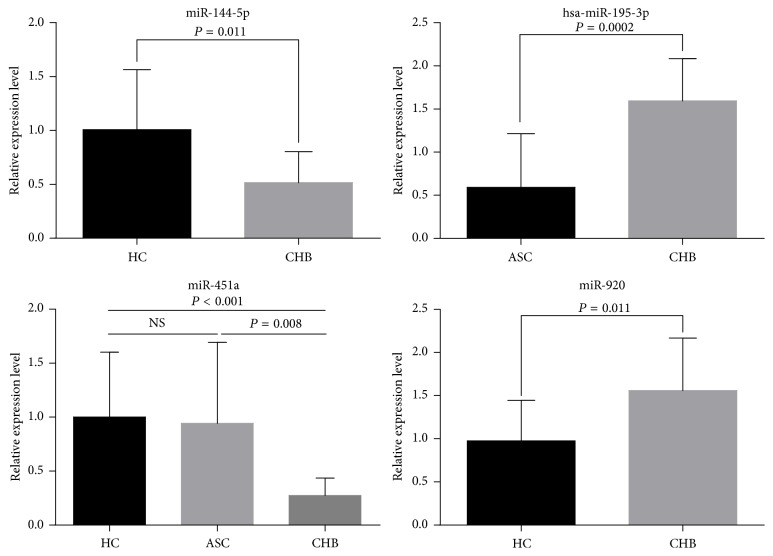
Validation of the microarray data by qRT-PCR analysis. Changes in the expression of the four randomly selected miRNAs showed good agreement between the RT-PCR and microarray results.

Four miRNAs with abnormal expression, namely, hsa-miR-195-3p, hsa-miR-144-5p, hsa-miR-451a, and hsa-miR-920, were selected and analyzed by real-time quantitative PCR in order to validate the microarray results. Consistent with the array data, the expression of these miRNAs was either upregulated or downregulated in the ASC group and CHB group in comparison with the HC group (Figure 1). The expression levels of hsa-miR-920 and hsa-miR-195-3p still exhibited more than a 1.5-fold increase in the CHB group when compared to the HC or ASC group (*P* < 0.05). Furthermore, the expression of hsa-miR-144-5p was still significantly downregulated in the CHB group, with around 2-fold difference (*P* < 0.05). The expression of hsa-miR-451a was similar between the HC and ASC groups, but it was significantly higher in the CHB group (*P* < 0.001 and *P* = 0.008, resp.).

### 3.4. Correlation of Four miRNAs with Clinical Indicators of ASC or CHB Patients

To investigate whether cellular miR-195-3p, miR-144-5p, miR-451a, and miR-920 were related to HBV infection, we correlated these four miRNAs' expression level with clinical indicators of HBV infection including serum ALT level, HBsAg level, and HBV DNA level. However, no significant correlation was found. Although the levels of miR-144-5p and miR-451a were slight negatively correlated with ALT (*r* = −0.329, −0.270; *P* = 0.135, 0.135), the difference was statistically insignificant.

### 3.5. GO Analysis of the Target Genes of the Differentially Expressed miRNAs

**Figure 2:**
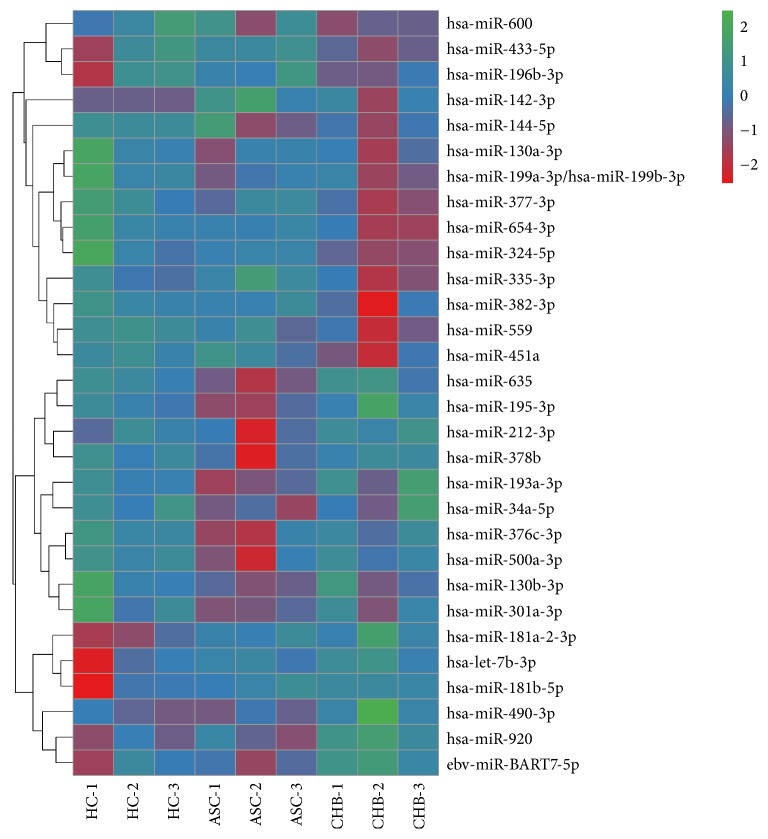
Hierarchical cluster analysis of the differentially expressed miRNAs. The red boxes represent upregulation of the corresponding miRNA, and the green boxes represent downregulation of the corresponding miRNA.

**Figure 3:**
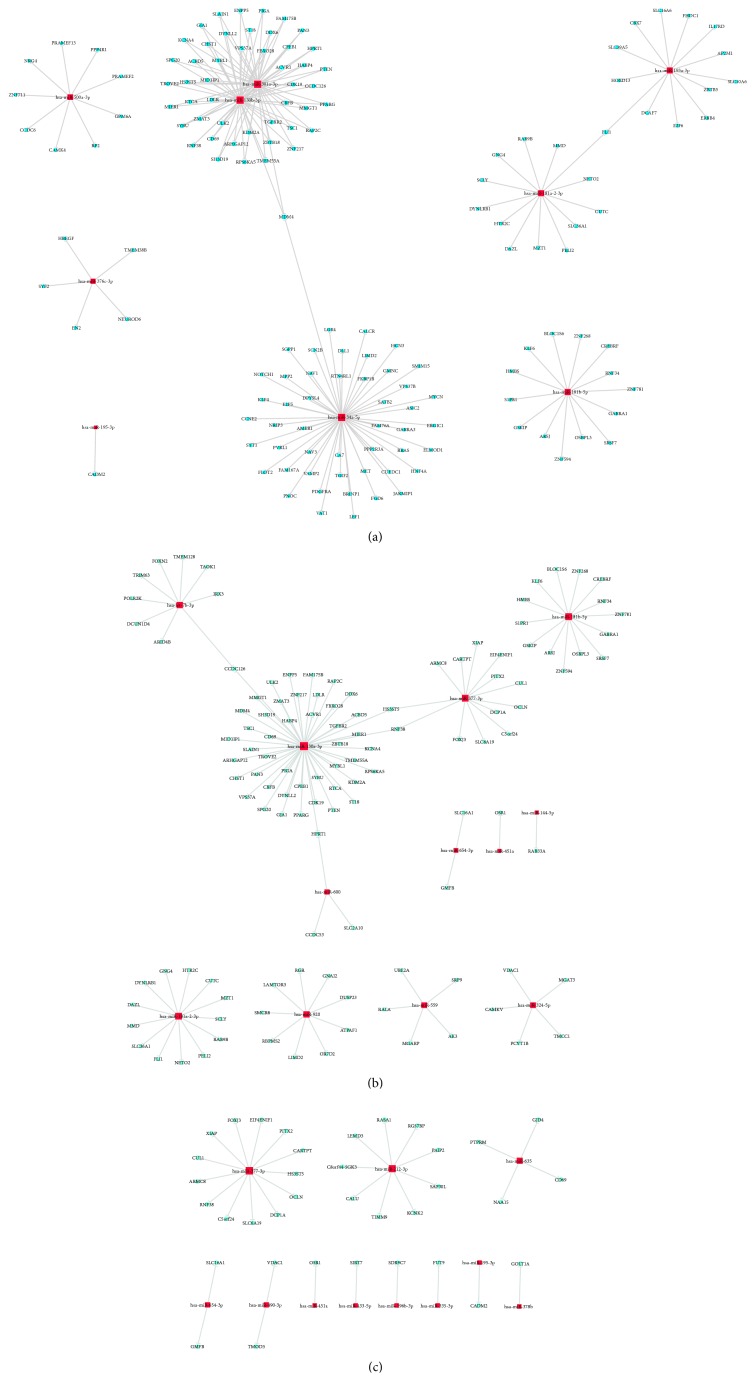
miRNA-gene networks built using the target genes of the differentially expressed miRNAs between the ASC group and HC group (a), CHB group and HC group (b), and CHB group and ASC group (c). The red squares represent miRNAs, and the blue round spots represent genes. The lines represent the interactions between miRNAs and genes.

**Figure 4:**
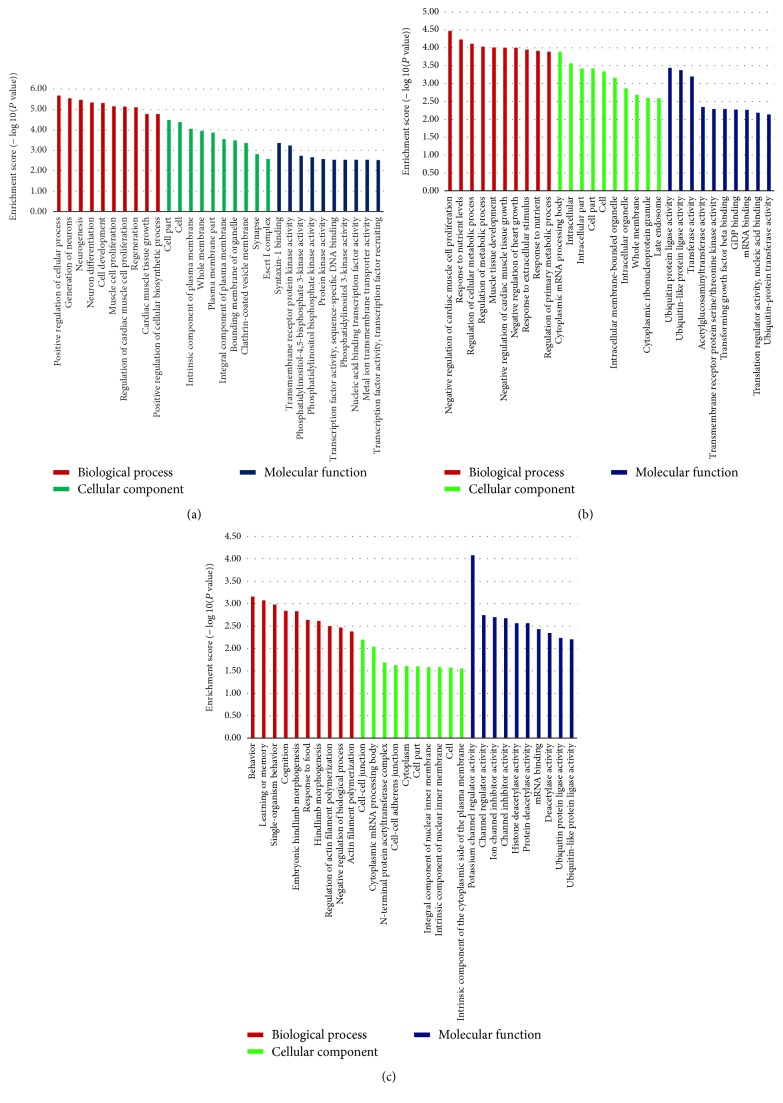
Functional classification of the target genes of differentially expressed miRNAs between the ASC group and HC group (a), CHB group and HC group (b), and CHB group and ASC group (c).

**Figure 5:**
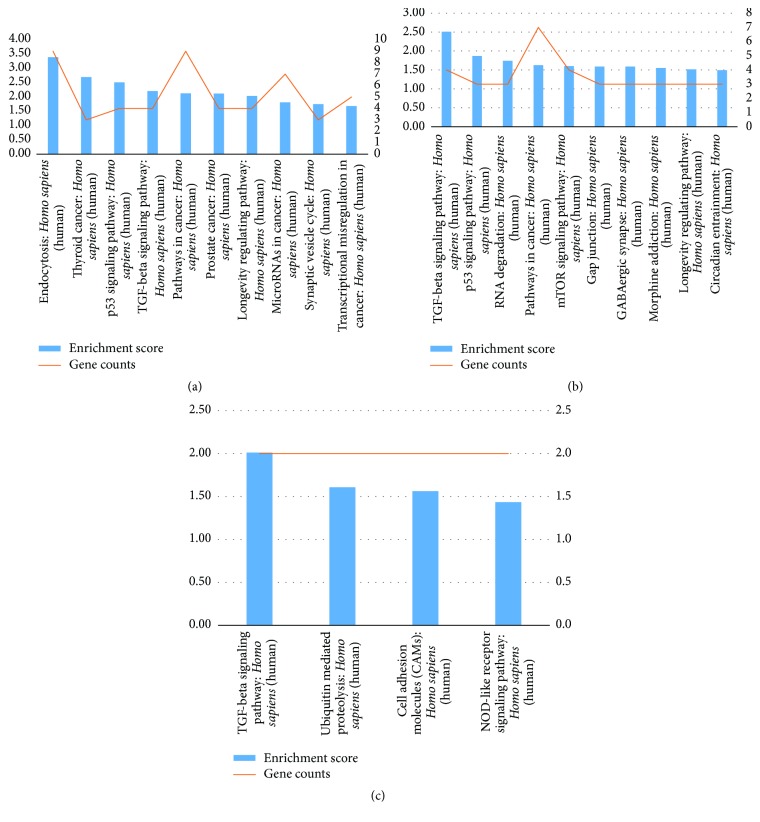
Pathway enrichment analysis of the target genes of differentially expressed miRNAs between the ASC group and HC group (a), CHB group and HC group (b), and CHB group and ASC group (c).

The sample size was rather small, and there were individual differences within each group. Therefore, some false positive results were obtained, as some miRNAs showed abnormally high or low expression only in certain samples. Therefore, we screened more valuable miRNAs for target gene prediction, GO analysis, or signaling pathway analysis (Table 4). Hierarchical cluster analysis was performed to analyze the remaining data (Figure 2). The target sites were predicted using targetscan7.1 and mirdbV5. The final target genes were those that were predicted by all the three miRNA prediction tools. miRNA-gene networks (Figure 3) based on the regulatory relationships between the miRNAs and their target genes were built. A total of 150 target genes were predicted for the differentially expressed miRNAs between the ASC and HC group; 119 genes, for the differentially expressed miRNAs between the CHB and HC group; and 36 genes, for the differentially expressed miRNAs between the CHB and ASC group. GO analysis consists of three components, namely, biological processes, cellular components, and molecular functions. The ten most commonly observed terms in each component were plotted to compare their differences between each group (Figure 4). The predicted target genes of the miRNAs that were differentially expressed in the ASC and CHB groups compared to the HC group were primarily involved with response to nutrient, regulation of primary metabolic process, cell part, kinase activity, and binding. Moreover, the predicted target genes of the miRNAs that were differentially expressed between the CHB group and ASC group were involved with learning or memory, cell-cell adherens junction, and ion channel inhibitor activity. Pathway enrichment analysis was performed to further understand the functions and signal pathways of these predicted gene targets (Figure 5). The results indicated that the target genes of differentially expressed miRNAs between the ASC group and HC group were related to 26 signaling pathways, particularly the pathways associated with endocytosis, thyroid cancer, and p53 signaling pathway. Further, the target genes of the differentially expressed miRNAs between the CHB group and HC group played a role in 12 signaling pathways, primarily the TGF-beta signaling pathway, p53 signaling pathway, and RNA degradation. Finally, the target genes of the differentially expressed miRNAs between the ASC group and CHB group were related to 4 signaling pathways that were primarily involved with TGF-beta, ubiquitin mediated proteolysis, and cell adhesion molecules (CAMs).

### 3.6. miRNA-GO/Pathway Network Analysis

**Figure 6:**
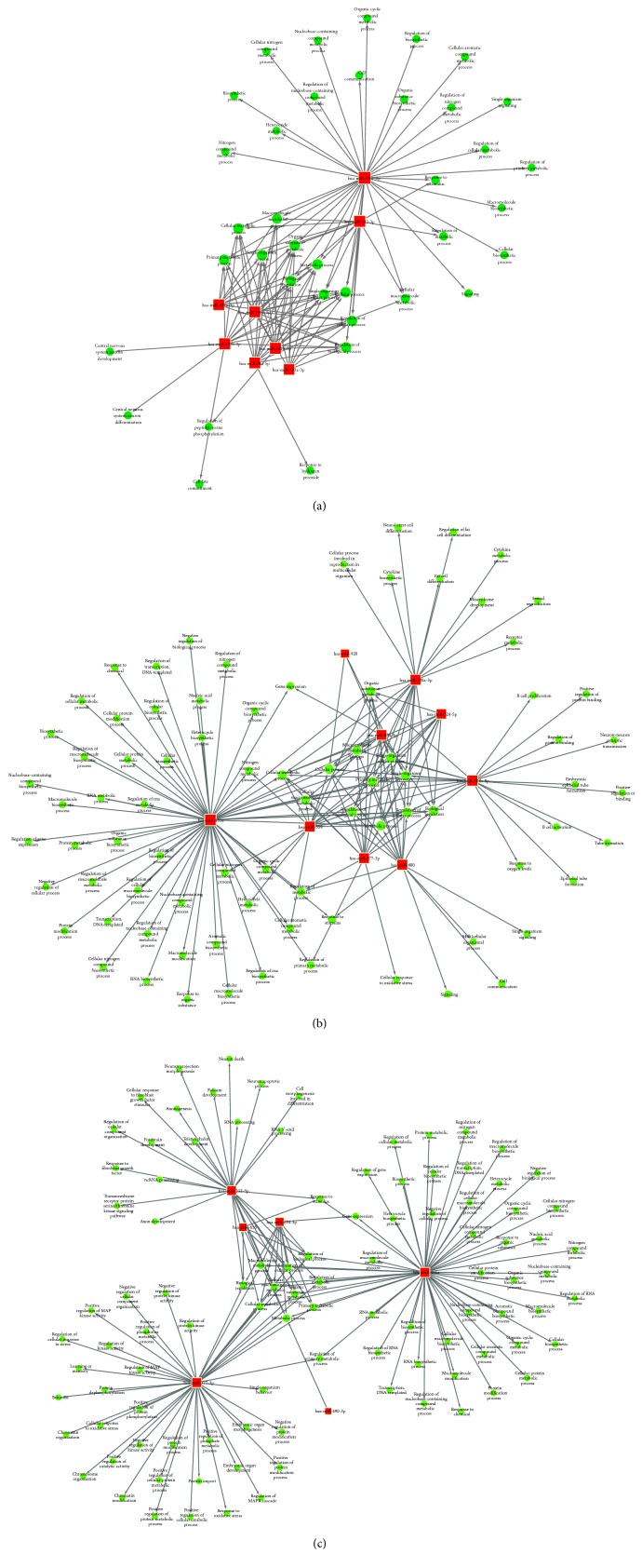
miRNA-GO networks based on the identified GO terms of the target genes of the differentially expressed miRNAs between the ASC group and HC group (a), CHB group and HC group (b), and CHB group and ASC group (c). The red boxes represent differentially expressed miRNAs; the blue circles represent the significant GO terms; and the straight lines represent interactions between the miRNA and the GO term. GO, gene ontology; miRNA, microRNA.

**Figure 7:**
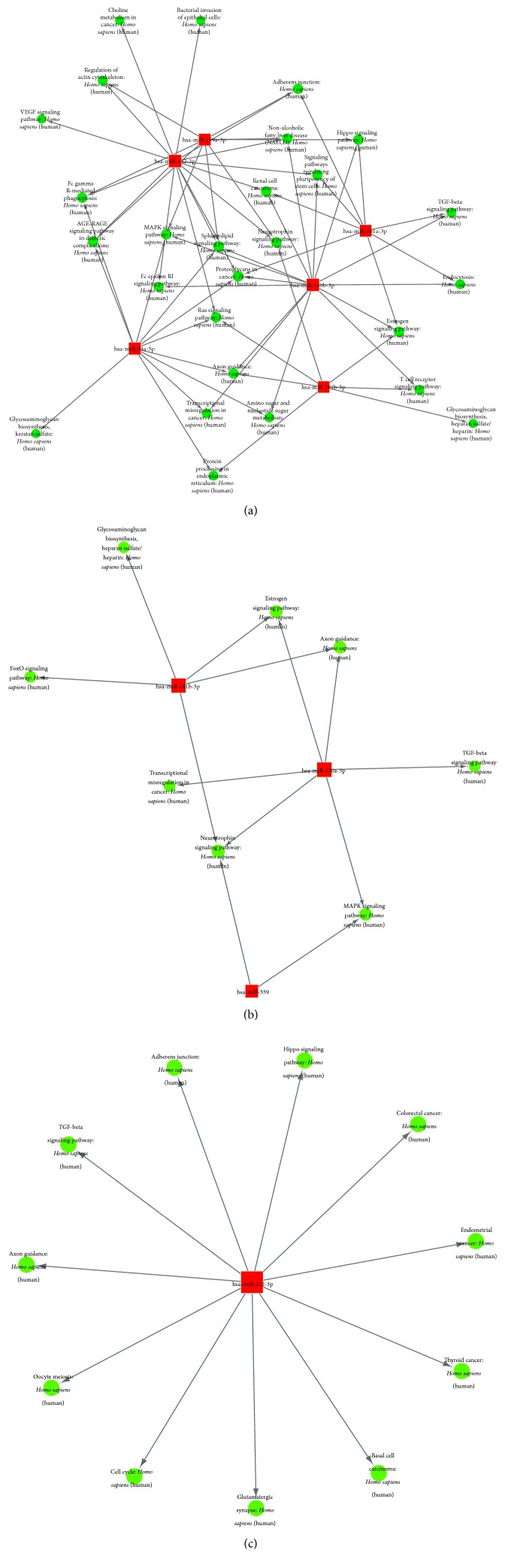
miRNA-pathway networks based on the identified pathways of the target genes of the differentially expressed miRNAs between the ASC and HC group (a), between the CHB and HC group (b), and between the CHB and ASC group (c). The red squares represent miRNAs; the blue round spots represent the pathways; and the lines represent interactions between the miRNAs and their target gene pathways.

To better understand the associations between the differentially expressed miRNAs and the results of GO and signaling pathway analyses, miRNA-GO networks (Figure 6) and miRNA-pathway networks (Figure 7) were built. Differentially expressed miRNAs, the GO terms that their target genes were linked to, and the pathways they were involved in according to the pathway enrichment analysis are represented as nodes in the graph, and the biological relationship between two nodes is represented as a line. All lines were supported by at least one study from the published literature, a textbook, or functional information in the GO or KEGG database. Based on the lines, we could then infer the key roles of the relationships depicted in the networks. The degree of complexity of each node was of primary concern, because the weight (importance) of the regulator (in this case, the miRNA) increases as the degree of complexity increases, according to the theory of network biology [24]. The visualized networks indicated that miR-142-3p, miR-181b-5p, miR-199a-3p, miR-130b-3p, miR-34a-5p, miR-301a-3p, and miR-376c-3p played regulatory roles in modulating the molecular networks in ASCs. Further, the networks demonstrated that miR-181b-5p, miR-130a-3p, miR-559, miR-920, miR-377-3p, miR-654-3p, miR-451a, miR-600, and miR-324-5p might be crucial regulators of pathogenesis in CHB patients. Finally, it was shown that miR-212-3p, miR-490-3p, miR-635, miR-377-3p, miR-654-3p, and miR-451a played a prominent role in the global signaling networks and pathways involved in the progression of chronic liver disease, as these miRNAs carried considerable weight in both the ASC and CHB groups.

## 4. Discussion

In this report, utilizing miRNA array, we analyzed the global miRNA expression profiles in the PBMCs of healthy control individuals and HBV-infected patients who were asymptomatic carriers or had chronic hepatitis B infection. Bioinformatics analysis of the microarray results revealed a set of miRNAs that were differentially expressed in the PBMCs of ASCs and CHB patients compared to the HCs. In addition, a significant number of the differentially expressed miRNAs were found in both ASCs and CHB patients. Moreover, we found that 144 mRNAs exhibited more than a 2-fold difference in their expression between the CHB group and ASC group. These observations indicated that common and phase-specific mechanisms may exist at the miRNA level in ASCs and CHB patients. Thus, investigating these miRNAs and their mechanisms might be of great importance for further research into the pathogenesis of hepatitis B infection.

miRNAs are likely to play a prominent role in altering the global signaling networks and pathways involved in the progression of liver disease. Therefore, in this study, we identified the target genes of the differentially expressed miRNAs and built miRNA-gene networks to study their interactions; further, we used GO and signaling pathway analysis to determine the functions and biological pathways of the target genes. GO analysis demonstrated that the target genes were primarily involved with response to nutrient, regulation of primary metabolic process, cell part, kinase activity, and binding, and pathway enrichment showed that the target genes were involved in several important pathways, such as those related to endocytosis, ubiquitin mediated proteolysis, TGF-beta signaling pathway, and p53 signaling pathway. Finally, the significance of the identified miRNAs was determined by building miRNA-GO and miRNA-pathway networks and calculating the degree of complexity of the nodes [57]. Using this analytical approach, we identified the key miRNAs at the centre of the signal transduction network in patients with HBV infection (both carriers and those with chronic infection). Since the remaining miRNAs also had a considerable degree of complexity, they will be the subject of our future studies. Meanwhile, our current results indicate that miR-142-3p, miR-181b-5p, miR-199a-3p, miR-130b-3p, miR-34a-5p, miR-301a-3p, and miR-376c-3p may be closely related to disease pathogenesis in ASCs and that miR-181b-5p, miR-130a-3p, miR-559, miR-920, miR-377-3p, miR-654-3p, miR-451a, miR-600, and miR-324-5p may play an important role in the pathogenesis of chronic hepatitis B. Further, miR-212-3p, miR-490-3p, and miR-635, and especially miR-377-3p, miR-654-3p, and miR-451a, may be associated with the progression of hepatitis B-related diseases, as they were found to be significant in both ASCs and CHB patients. Several of these miRNAs have already been reported in previous studies, namely, miR-181b-5p, miR-199a-3p, miR-130b-3p, miR-34a-5p, miR-301a-3p, miR-130a-3p, miR-654-3p, miR-451a, miR-377-3p, and miR-600. However, miR-142-3p, miR-376c-3p, miR-559, miR-920, miR-324-5p, miR-212-3p, miR-490-3p, and miR-635 have not been previously reported in studies on HBV-related diseases, which makes this finding novel. No significant correlation was found between the levels of miR-195-3p, miR-144-5p, miR-451a, and miR-920 and clinical indicators of HBV infection. It is likely due to the small sample size of this study and clinical information missed in some patients. Taking the correlated between miR-144-5p, miR-451a, and ALT as an example, it was possible that the association was real; with a larger sample, we might find that the association did become statistically significant. In attempts to answer these questions, we summarized miRNAs that was reported as a strong correlation with clinical indicators of HBV infection (Table 5).

The functions of the previously reported miRNAs are described here. Previous studies have demonstrated that miRNA-181a is a critical player in the modulation of both innate [58] and adaptive [59] immunity and is involved in distinct pathological processes. miR-199a-3p has been reported to bind to and directly suppress HBV RNA [60]. miR-130a and miR-130b share the same seed sequences, and miRNA-130a can inhibit hepatitis B virus replication by targeting PGC1*α* and PPAR*γ* [51]. Moreover, downregulation of miR-377 contributes to IRX3 deregulation in hepatocellular carcinoma [61]. miRNA-34is associated with hepatic fat metabolism [62]. A recent study has shown that the expression of miR-301 in the serum was significantly higher in HCC patients than in healthy subjects [63]. Finally, has-miR-600 was found to be associated with inflammatory and cell cycle pathways [12]. In general, miRNAs could act as a cellular anti-viral defense, as siRNAs do in plants and lower eukaryotes, or cellular miRNAs could be exploited by the virus to help establish a favorable environment for its replication and survival. As shown in Table 6, we summarized miRNAs that was reported as a dysregulated miRNA by HBV infection.

In summary, we have comprehensively analyzed the cellular miRNA profiles of HBV-infected carriers and chronic disease patients. We have identified certain important miRNAs that were differentially expressed in the patients and were also differentially expressed between the carriers and chronic disease patients. Further, network biology analysis of the target genes of these miRNAs demonstrated the global regulation of signaling network pathways by these miRNAs. Further studies on the molecular regulating mechanisms of miRNAs in HBV-related diseases need to be conducted to further elucidate the pathogenesis of chronic hepatitis B and identify therapeutic targets for treatment.

## Figures and Tables

**Table 1:** Primer sequence of quantitative RT-PCR.

Gene name	Primer sequence
U6	F: 5′-GCTTCGGCAGCACATATACTAAAAT-3′ R: 5′-CGCTTCACGAATTTGCGTGTCAT-3′
hsa-miR-195-3p	F: 5′-CCAATATTGGCTGTGCTGCTC-3′
hsa-miR-144-5p	F: 5′-CGCGGATATCATCATATACTGTAAG-3′
hsa-miR-451a	F: 5′-CGAAACCGTTACCATTACTGAGTT-3′
hsa-miR-920	F: 5′-GGGGAGCTGTGGAAGCAGT-3′

**Table 2:** Clinical data of patients used for cellular miRNA analysis.

Clinical characteristics	HC	ASC	CHB	*P* value
Number of patients	16	16	16	*P* = NS
Sex number (%)				
Female	6	5	6	
Male	10	11	10	*P* = NS
Age (years)				
Median	45	43	42	
Range	34–65	27–64	25–62	*P* = NS
ALT				*P* = NS (ASC versus HC )
Median	27.43	34.38	95.36	*P*< 0.001 (CHB versus HC)
Range	9–69	17–89	16–253	*P*< 0.001 (CHB versus ASC)
AST				*P* = NS (ASC versus HC)
Median	24	35.29	75.50	*P* = 0.028 (CHB versus HC )
Range	14–34	21–84	9–327	*P* = NS (CHB versus ASC )
HBV DNA				
Median	NA	1.51 × 10^6^	2.70 × 10^8^	
Range	NA	2.12 × 10^3^–5.99 × 10^6^	2.37 × 10^4^–2.51 × 10^9^	*P* = NS (CHB versus ASC)
HBsAg				
HBsAg (+)	NA	4 (25%)	10 (62.5%)	
HBsAg (−)	NA	12 (75%)	6 (37.5%)	*P* = 0.033 (CHB versus ASC)

**Table 3:** Common miRNAs differentially expressed between ASC group and CHB group.

	CHB group	
	Up	Down
ASC group		
Up	miR-181a-2-3p, miR-876-5p, miR-3184-3p, miR-4711-3p, miR-181b-5p	
Down		miR-151a-5p, miR-330-5p, miR-130a-3p, miR-718, miRPlus-B1114, miR-326, miR-151a-3p, miR-139-5p, miR-369-3p, miR-33a-5p, miR-33b-5p, miR-3679-3p, miR-708-5p, miR-199a-3p, miR-190a-5p, miR-335-5p, miR-122-3p, miR-376a-5p, miR-495-3p, miR-498, miR-K12-8-5p, miR-22-3p, miR-126-3p, miR-1915-3p, miR-4328, miR-106b-5p, miR-331-3p, miR-151a-5p, miR-199a-5p, miR-339-5p, miR-143-3p, miR-652-3p, miR-4723-3p

**Table 4:** Significant differentially expressed miRNA in each group.

	ASC versus HC	CHB versus HC	CHB versus ASC
Upregulation	hsa-miR-181a-2-3p, hsa-miR-142-3p hsa-miR-181b-5p	hsa-miR-181a-2-3p hsa-miR-920 hsa-let-7b-3p hsa-miR-181b-5p	hsa-miR-490-3p hsa-miR-212-3p hsa-miR-635 ebv-miR-BART7-5p hsa-miR-195-3p hsa-miR-378b
Downregulation	hsa-miR-376c-3p hsa-miR-199a-3p hsa-miR-130b-3p hsa-miR-193a-3p hsa-miR-195-3p hsa-miR-500a-3p hsa-miR-34a-5p hsa-miR-301a-3p	hsa-miR-130a-3p hsa-miR-382-3p hsa-miR-559 hsa-miR-377-3p hsa-miR-654-3p hsa-miR-451a hsa-miR-600 hsa-miR-144-5p hsa-miR-324-5p	hsa-miR-335-3p hsa-miR-433-5p hsa-miR-377-3p hsa-miR-654-3p hsa-miR-451a hsa-miR-196b-3p

**Table 5:** Correlation of miRNAs and clinical indicators of HBV infection.

MicroRNA	Relevance	Clinical indicators	Citation
miR-122	Positive correlation	HBV DNA	[16]
	Positive correlation	ALT levels	[17]
	Positive correlation	HBsAg levels, ALT levels, and HBV DNA titers	[18]
miR-22	Positive correlation	HBsAg levels, ALT levels	[18]
miR-29	Positive correlation	HBV DNA	[16]
	Negatively correlated	Liver fibrotic stages and necroinflammation grades	[19]
miR-210	Positive correlation	HBV DNA, HBs antigen, alanine aminotransferase (ALT), and HAI score	[20]
miR-33a	Positive correlation	Hepatic fibrosis	[21]
miR-125b	Negatively correlated	HBV DNA intermediates and secretion of HBsAg and HBeAg	[22]
miR-146a	Positive correlation	HBsAg levels	[23]
	Positive correlation	ALT	[14]
miR-548ah-5p	Negative correlation	HBV DNA	[14]

**Table 6:** Deregulated miRNA in HBV infection or HBV-related disease.

MicroRNA	Alteration	Target	Citation
miR-1	Up	HDAC4, FXRA	[25]
miR-15a	Down	HBp and HBx	[26]
miR-15b	Down (early stage) Up (late stage)	HNF1*α*	[27]
miR-15a/16	Down	Bcl-2	[28]
miR-17	Up	ULK1, ATG7, p62	[29]
miR-17-92 cluster	Up	HBV transcripts, E2F1	[30, 31]
miR-18a	Up	ESR1, ERa	[29, 32]
miR-19b	Up	P53	[30]
miR-20a	Up	Egln3/PHD3	[30]
miR-21	Up	PDCD4, PTEN	[33]
miR-22	Down	CDKN1A, AKT3, p21	[34, 35]
miR-26b	Down	CHORDC1	[36]
miR-27a	Up	MAP2K4, TR*β*1	[30]
miR-29a	Up	PTEN	[37]
miR-29c	Down	TNFAIP3	[38]
miR-33a	Up	Smad7	[21]
miR-34c	Down	TGIF2	[39]
miR-93	Down	MICA	[40]
miR-99a	Down	AGO2	[41]
miR-101	Down	FOXO1, EZH2, DNMT3A	[42–44]
miR-103	Up	PER3, CDK5R1	[29]
miR-106a	Up	P130, FAS	[29]
miR-107	Up	CDK8, let-7	[29]
miR-122	Down	HBV DNA polymerase, Cyclin G1, HO-1, NDRG3, PPAR*γ*, PBF	[45–47]
miR-125a-5p	Up	HBsAg	[46, 48]
miR-125b	Down	SCNN1A	[22]
miR-125b-5p	Up	HBsAg, LIN28B/let-7 axis	[49, 50]
miR-126	Down	PI3KR2, Crk, PLK2	[30]
miR-130a	Down	PPARG, Era, PGC1*α*, PPAR*γ*	[30, 45, 51]
miR-141	—	PPAR*α*	[46]
miR-143	Up	FNDC3B	[47]
miR-145	Down	CUL5	[52]
miR-146a	Up	STAT1, CFH, RIG-I, RIG-G	[23, 46, 53]
miR-148a	Up	c-Met, Wnt	[30]
miR-152	Down	DNMT1	[46]
miR-155	Up	C/EBP, SOCS1	[46]
miR-181a	Up	HLA-A, E2F5	[46, 47]
miR-199a	Up	HBsAg	[46]
miR-205	Down	HBx	[46]
miR-210	Up	HBV pre-S1	[46]
miR-221	Up	PI3-K/Akt	[29]
miR-224	Up	HOXD10, CDC42, Smad4	[29, 47]
miR-331-3p	Up	ING5	[54]
miR-370	Down	NFIA	[45]
miR-372/373	Up	NFIB	[46]
miR-429	Down	NOTCH1	[55]
miR-449a	Up	CREBFXRA	[45]
miR-501	Up	HBXIP	[46]
miR-545/374a	Up	ESRRG	[29]
miR-548ah	Up	INF*γ*R1	[46]
miR-581	Down	Dicer, EDEM1	[46]
miR-602	Down	RASSF1A	[29]
miR-939	Down	CEBPA	[45]
miR-1231	Up	HBcAg	[46]
miR-4717	Down	PD-1	[56]
Let-7a	Down	CCR7	[29]
